# Bio-efficacy of *Mangifera* leaf extracts on mortality of *Aedes aegypti* and inhibition of egg hatching

**DOI:** 10.14202/vetworld.2022.1753-1758

**Published:** 2022-07-23

**Authors:** Nur Mahdi, Muhammad Rasyid Ridha, Deni Setiawan, Muhammad Riki Shindi Praristiya, Nita Rahayu, Bayu Purnama Atmaja

**Affiliations:** 1 Pharmacy Program, College of Health Darul Azhar, Tanah Bumbu, South Kalimantan, Indonesia; 2 Organization Research for Health, The National Research and Innovation Agency (BRIN-Indonesia), Cibinong, West Java, Indonesia; 3 Pharmacy Program, Lambung Mangkurat University, Banjarbaru, South Kalimantan, Indonesia; 4 Nurse Program, College of Health Darul Azhar, Tanah Bumbu, South Kalimantan, Indonesia

**Keywords:** *Aedes aegypti*, bio-efficacy, leaf extracts, *Mangifera*

## Abstract

**Background and Aim:**

To develop an environmentally friendly alternative to mosquito larvicides for vegetables, leaf extracts of *Mangifera laurina*, *Mangifera casturi*, *Mangifera indica*, *Mangifera odorata*, *Mangifera caesia*, and *Mangifera foetida* were prepared. This study aimed to determine the biological efficacy of several *Mangifera* leaf extracts on the mortality of *Aedes aegypti* mosquito and the inhibition of egg hatching.

**Materials and Methods:**

Extraction was performed in an organic solvent (methanol) using a Soxhlet extractor. The larvicidal potential of six leaves of *Mangifera* essential oil was evaluated against the third instar larvae of *A. aegypti* at concentrations of 1500, 2000, 3000, and 5000 ppm using the World Health Organization protocol. After Probit analysis, the 48 h LC_50_ and LC_90_ values of the essential oils were determined. The inhibitory effect on egg hatching was also tested at 160, 320, 480, and 640 ppm.

**Results:**

The extraction of essential oils from several *Mangifera* species had excellent larvicidal activity and inhibitory activity against *A. aegypti* egg hatching. The LC_50_/LC_90_ values were: *M. casturi*, 241/1964 ppm; *M. laurina*, 2739/4035 ppm; and *M. caesia*, 1831/2618 ppm. The inhibitory effect on hatching was 78% for *M. foetida*, 70% for *M. caesia*, and 59% for *M. casturi*.

**Conclusion:**

The test results indicate the potential of some *Mangifera* species for use as larvicides and inhibitors of egg hatching; thus, they have the potential to control *A. aegypti* in the early stages of development.

## Introduction

The mosquito *Aedes aegypti* Linn. is known as the main vector of dengue, Zika, and chikungunya fever worldwide. This mosquito can be found in tropical and subtropical climates worldwide and is generally found in urban and semi-urban areas [1]. The number of cases of dengue fever in the world has reached 100–400 million every year [2]. Another study on the prevalence of dengue hemorrhagic fever estimates that 3.9 billion people are at risk of being infected with dengue virus [3], and the relative contribution to imported cases of dengue fever worldwide is 76.3% from Asia and 15.7% from America [4].

One of the larval controls that have been applied in the community is the use of chemical insecticides, namely, synthetic larvicides. Although synthetic larvicides have been used since 1976, any insecticides are harmful to humans because the ingredients contain synthetic compounds and are dangerous when the appropriate dose is exceeded [5]. The use of synthetic insecticides, especially larvicides, result in several side effects, including insect resistance, environmental pollution, and insecticide residues [6]. The use of vegetable larvicides has now been tested again to prevent the occurrence of resistance.

*Mangifera* is the name of a genus in the Mango tribe (or Anacardiaceae). It comprises approximately 35–40 types of mangoes that are spread throughout tropical Asia, especially in the biogeographical area of Malaysia [7]. One species, *Mangifera indica*, has been grown in production gardens in various tropical regions. The island of Borneo has the highest number of species, approximately 31, so it is estimated to be a center of species diversity [8].

Kalimantan Mango (*Mangifera casturi*) and Binjai plant (*Mangifera caesia*) are typical plants of Kalimantan that are easy to find [8]. Phytochemical tests show that Binjai leaves contain secondary metabolites in the form of saponins, tannins, triterpenoids, steroids, alkaloids, and flavonoids [9]. Some of these substances are known to be present in larvicidal essential oils [10]. Another study used the aqueous extract of the leaves of *M. indica* L. (Anacardiaceae) with nanoparticles of titanium dioxide and found that they were effective against larvae of *R. microplus*, *Hyalomma anatolicum*, *Haemaphysalis bispinosa*, *Anopheles subpictus*, and *Culex quinquefasciatus*; the LC_50_ values were 28.56, 33.17, 23.81, 5.84, and 4.34 mg/L, respectively [11]. In other studies, *M. indica* leaves were antimicrobial (against *Salmonella* Typhi, *Klebsiella pneumonia*, *Enterobacter aerogens*, *Mycobacterium tuberculosis*, *Streptococcus pyrogens*, *Pseudomonas aeruginosa*, *Proteus vulgaris*, *Escherichia coli*, and *Staphylococcus aureus*) and biolarvicidal in raw fruit [12].

This study aimed to determine the bioactivity of six *Mangifera* leaf extracts on the mortality of *A. aegypti* and the inhibition of eggs hatching. Once proven effective, the fraction can be used in the community to control larval growth and egg hatchability, which will control dengue transmission.

## Materials and Methods

### Ethical approval

Ethical approval was obtained from the Ethics committee of College of Health Darul Azhar, Tanahs Bumbu, Indonesia, number EA.006.10/11/STIKES-DA/2021.

### Study period and location

The study was conducted from February 2021 to July 2021. Plants extraction and maceration of Simplicia were performed in Herbarium Laboratory College of Health Darul Azhar. Tests for larvae and eggs of *A. aegypti* were conducted in Entomology Laboratory, Health Research and Development Agency, Tanah Bumbu Unit, Ministry of Health, Republic of Indonesia.

### Plant material

Hampalam Folium (*Mangifera laurina*), Kasturi Folium (*M. casturi*), Mangga Folium (*M. indica*), Kuwini Folium (*Mangifera odorata*), Binjai Folium (*M. caesia*), and Hambawang Folium (*Mangifera foetida*).

The plant samples were collected from Palangka Raya University: Herbarium Center for International Co-operation in Management of Tropical Peatland (CIMTROP), at an altitude of 75–100 m above sea level. Clean mature leaves were collected and taken to the College of Health, Darul Azhar, for processing and identification, and voucher specimens were kept at the Palangka Raya University: Herbarium CIMTROP.

### Mosquito test

The larvae and eggs of *A. aegypti* used in this study came from the colony laboratory, cultured at room temperature (25–28°C) under a 12 h light: 12 h dark in the insectarium of the Entomology Laboratory, Health Research and Development Agency, Tanah Bumbu Unit, Ministry of Health, Republic of Indonesia. The population used is the third and fourth instar larvae collected using a pipette. The eggs used were from hatching on the same day and in good condition and were examined with a dissecting microscope at a magnification of 10×.

### Preparation of extracts

The extraction of each plant (*M. laurina*, *M. casturi*, *M. indica*, *M. odorata*, *M. caesia*, and *M. foetida*) was performed using a maceration method, in which the material is soaked in methanol for 3 × 24 h and occasionally stirred until liquid extract is obtained. Furthermore, the liquid extract is evaporated until the crude extract is obtained.

### Larvicidal bioassay

The larvicidal and ovicidal potential of ethanolic extracts of numerous leaves toward *A. aegypti* mosquitoes were evaluated following the World Health Organization protocol to check for mosquito mortality, but with mild modifications [13]. They observed the instar level three larvae because these larvae are exceedingly robust to the effect of outside factors and have a good survival rate (SR). The SR of the aquatic section of *A. aegypti* reports a susceptible duration from the primary instar to the second instar section.

In total, 4375 *Aedes* larvae were used in the study; 25 larvae were used at each concentration (1500, 2000, 3000, and 5000 ppm), with four replicate measurements were taken. The larval testing experiment used a plastic container with a total volume 150 mL and introduced extract and water. Observations were made at 5, 10, 15, 30, 60, 120, 180, 300, 360, 720, 1440, and 2880 min.

After exposure for 48 h, larval mortality was recorded. The controls used were one part acetone in water, 1% ethanol in water, and water only. For the positive control, the mosquito larvicide Abate 1SG (Abate 500E, liquid pay attention containing 500 g of organophosphates) was used.

### Ovicidal bioassay (hatchability)

The ovicidal activity was determined by measuring egg hatchability. *A. aegypti* eggs used in the test came from laboratory colonies, bred at room temperature (25–28°C) in an insectarium at the Insectarium of the Entomology Laboratory of the Indonesian Institute for Soil Research and Development. Freshly laid eggs on paper strips were observed under a stereomicroscope for survival. Then, 20–30 viable eggs were placed for each stock solution concentration. The viable eggs were exposed to different soluble crude ethanol extract doses and the soluble ethyl acetate, hexane, and water fractions. The test was performed four times with concentrations of 160, 320, 480, and 640 ppm. Negative control cups containing 1% acetone in water, 1% ethanol in water, and water alone were stored separately. Egg hatchability at 72 h post-treatment was observed, and the data were recorded.

### Statistical analysis

The percentage of larval mortality was calculated by dividing the number of dead and inactive larvae by the total number of larvae that were tested multiplied by 100. Microsoft Excel and IBM SPSS Statistics for Windows software were used for data analysis. The Pearson fit test was used to determine whether Probit’s model adequately fit the data provided by the experiment. LC_50_ and LC_90_ with a 95% confidence level were presented. The percentage of egg hatchability at each dose was calculated by dividing the number of eggs hatched by the total number of eggs inserted, multiplied by 100. The average hatchability at each dose was calculated, including the standard deviation. One-way analysis of variance was used to determine whether there was a significant difference in the mean hatchability between the different doses.

## Results and Discussion

The activity of six *Mangifera* leaf extracts on the killing and hatchability of *A. aegypti* after 48 h of exposure in larvae and 72 h in eggs is presented in Table-1. Mortality increased with increasing concentration; in the negative control, no death was found; and in the positive control group, all larvae died. *M. casturi* has better efficacy at a concentration of 2000 ppm, compared with *M. caesia* and *M. laurina* at 5000 ppm. Likewise, for the hatching rate of eggs, the ability to inhibit hatching was accompanied by an increase in concentration. *M. odorata*, *M. laurina*, and *M. indica* showed stronger inhibition, that is, only 36% and 47% of eggs hatched, respectively, whereas *M. foetida* was only able to inhibit approximately 89% (Table-1). The increased concentration caused an increase in the death of larvae, and it was confirmed in research that the higher the concentration level, the higher the existing poison content [14]. Consequently, the larval paralysis process was faster because *Mangifera* extract can inhibit the feeding ability of the larvae and suppress the activity of the nervous system of larvae [15].

**Table 1:** Mean percentage response of Larva mortality and Eggs hatchability *Aedes aegypti* to various concentrations *Mangifera* leaf extracts.

Mean percentage response	Dose, ppm	*Mangifera foetida*	*Mangifera caesia*	*Mangifera odorata*	*Mangifera laurina*	*Mangifera casturi*	*Mangifera indica*
% Larva mortality after 48 h	1500	7.00 ± 0.95	75 ± 2.06	19 ± 0.50	76 ± 0.81	94 ± 2.38	21 ± 1.25
	2000	10 ± 0.57	87 ± 1.50	27 ± 2.21	87 ± 1.25	100 ± 1.70	14 ± 0.57
	3000	46 ± 2.38	99 ± 0.50	25 ± 0.95	89 ± 0.95	100 ± 1.6	14 ± 1.29
	5000	78 ± 1.73	100 ± 0.00	44 ± 1.41	100 ± 0.00	100 ± 2.06	23 ± 1.25
	Negative Control	0	0	0	0		0
Eggs hatchability after 72 h	160	100 ± 0.00	100 ± 0.00	73 ± 2.36	74 ± 0.57	84 ± 0.81	100 ± 0.00
	320	92 ± 0.81	100 ± 0.00	53 ± 1.89	57 ± 0.95	78 ± 1.29	100 ± 0.00
	480	89 ± 0.95	81 ± 0.50	44 ± 1.41	62 ± 0.57	71 ± 0.95	88 ± 0.81
	640	78 ± 1.29	70 ± 3.31	36 ± 2.16	47 ± 0.95	59 ± 0.95	47 ± 0.95
	Negative Control	100 ± 0.00	100 ± 0.00	98 ± 1.00	100 ± 0.00	100 ± 0.00	100 ± 0.00

**Figure-1:**
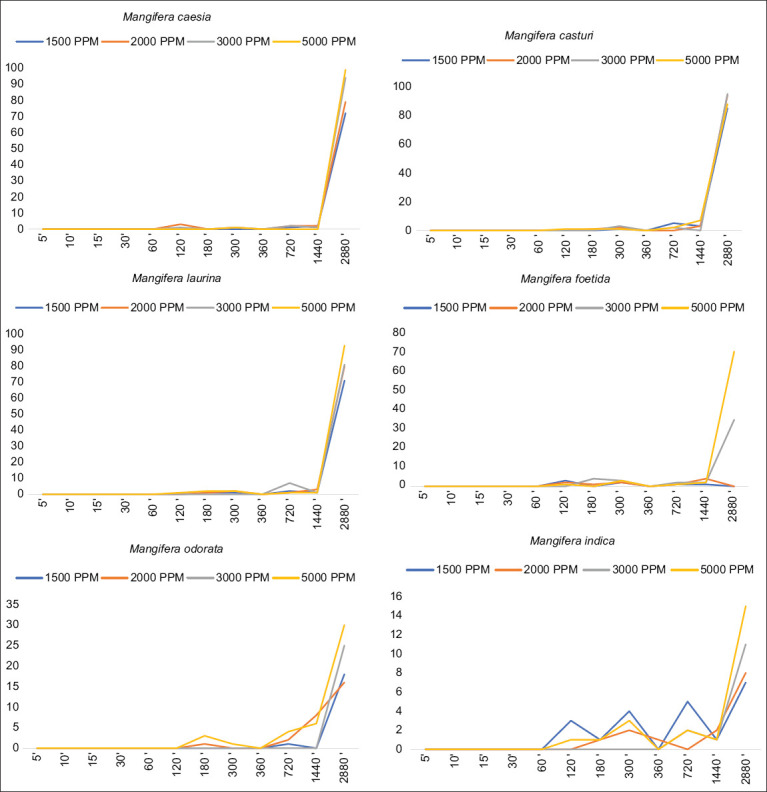
Larval death time in 48 h test on several *Mangifera* leaf extracts.

The larval death time within 48 h of the test is shown in Figure-1. The fastest death time was *M. indica*, starting at 60′′, then *M. foetida* 70′′, *M. caesia* 90′′, *M. odorata* 120′′, *M. casturi* 180′′, and finally *M. laurina* 360′′. The different times are probably because the stomach poison works by different species, even though the amount of poison that penetrates the digestive tract is the same [16]. Furthermore, Jayaraj *et al*. [17] states that the toxicity of insecticides in a species is influenced by the high and low levels of chemical compounds of the insecticide in the body of the target species.

*Mangifera* spp. are generally scattered in lowland forest areas (0–1000 m above sea level) and primary and secondary forests [8]. *M. casturi* is a species endemic to Borneo [18], and its potential as a biolarvicide has not been explored. The results of this study showed a very good ability as a biolarvicide. *M. casturi* contains essential oils containing terpenoids, steroids, and saponins [19]. Kasturi plant is known to be used as a traditional medicine because of flavonoid compounds [20]. Several study results show that flavonoid compounds, terpenoids, steroids, and saponins have antibacterial activity [21].

The lowest LC_50_ and LC_90_ concentrations were found for *M. casturi* at 241 and 1964 ppm, *M. caesia* at 1831 and 2618 ppm, and *M. laurina* at 2739 and 4035 ppm (Table-2). The lower the LC_50_ value of a substance, the higher the activity in killing experimental animals. Because of these substances, lower concentrations are needed to kill experimental animals over the same time period [22]. This study also showed that *M. caesia* and *M. laurina* could kill up to 100% larvae at a concentration of 5000 ppm. Both species are known to contain saponins, tannins, phenolics, and flavonoids. Tannins are toxic to insects and these compounds bind to proteins in the salivary glands and reduce the activity of digestive enzymes, reducing the rate of growth and nutritional disturbances [21]. This disturbance causes the death of larvae, and as reported by Chowdhury *et al*. [23] in 2008; tannins from extracts of *Eclipta prostrate*, *Hemidesmus indices*, and *Gymnema sylvestre* caused the death of *C. quinquefasciatus* larvae. Saponins are detergent-like substances that can damage the membrane, which will disrupt the lipid layer of the epicuticle and disrupt the endocuticular protein layer so that toxic compounds can easily enter into the larvae body [24]. The previous study conducted by Jawale [25] reported that saponins from the extract of *Sapindus mukorossi* stem, fruit extract of *Cestrum nocturne*, and leaf extract of *Cestrum diurnum* at 60 ppm resulted in 100% mortality of *A. aegypti*.

**Table 2:** Larvacidal activity and Egg hatchability of different leaf extracts of against *Aedes aegypty.*

Consentrationa (ppm)	Leaf extract					

	*Mangifera foetida*	*Mangifera caesia*	*Mangifera odorata*	*Mangifera laurina*	*Mangifera casturi*	*Mangifera indica*
Mortality ± SD						
1500	1.75 ± 0.9	18.7 ± 2.0	4.75 ± 0.5	19.0 ± 0.8	23.5 ± 2.3	5.25 ± 1.2
2000	2.50 ± 0.5	21.7 ± 1.5	6.75 ± 2.2	21.7 ± 1.2	25.2 ± 1.7	3.50 ± 0.5
3000	11.5 ± 2.3	24.7 ± 0.5	6.25 ± 0.9	22.2 ± 0.9	25.0 ± 0.0	3.50 ± 1.2
5000	19.5 ± 1.7	25.0 ± 0.0	11.0 ± 1.4	25.0 ± 0.0	25.0 ± 0.0	5.75 ± 1.2
Control	0.0 ± 0.0	0.0 ± 0.0	0.0 ± 0.0	0.0 ± 0.0	0.0 ± 0.0	0.0 ± 0.0
LC_50_	3415 (2988–3771)	1831 (412–2330)	6974 (6471–8110)	2739 (2638–2844)	241 (197–295)	237719 (171945–328656)
LC_90_	6175 (5405–7723)	2618 (1713–3349)	14978 (12942–16122)	4035 (3748–4107)	1964 (1316–2162)	6467977 (3438410–7534370)
% Hatchability ± SD						
160	25 ± 0.0	25 ± 0.0	18.2 ± 2.3	15.5 ± 0.5	21 ± 0.8	25 ± 0.0
320	23 ± 0.8	25 ± 0.0	13.2 ± 1.8	14.2 ± 0.9	19.5 ± 1,2	25 ± 0.0
480	22.2 ± 0.9	20.2 ± 0.5	11 ± 1.4	11.7 ± 0.9	17.7 ± 0.9	22 ± 0.8
640	19.5 ± 1,2	17.5 ± 3.3	9 ± 2.1	18.5 ± 0.5	14.7 ± 0.9	11.7 ± 0.9
Control	25 ± 0.0	25 ± 0.0	24.5 ± 1.0	25 ± 0.0	25 ± 0.0	25 ± 0.0

SD=Standard deviation

Concerning the effect on egg hatchability, leaf extracts of *M. odorata*, *M. laurina*, and *M. indica* resulted in strong egg hatching inhibitory activity and enhanced the larvicidal activity of the essential oils of *M. odorata*, *M. laurina*, and *M. indica*. Another similar study on *Cassia fistula* with methanol as a solvent can also be used as an egg hatching inhibitor [26]. In our current study, the mode of action of egg hatching inhibition and the larvicidal activity of essential oils were not studied, but the previous study conducted by Mathew *et al*. [27] have shown that mosquito eggshells are composed of several layers to protect the embryo, as well as chitin. The action of lipophilic substances on eggs is possible through the eggshell and disturbances in water and gas exchange [28] or by penetration into the egg. It can inhibit enzymatic reactions and hormonal activities that interfere with embryogenesis [29]. The mechanism of action of larvicides may be through essential oils, which can increase the tendency of tracheal flooding and chemical toxicity to mosquito larvae, or compounds in essential oils can interfere with the mitochondrial proton transfer process, causing larvae to die [30].

This study found that the essential oils of the leaves of *M. casturi*, *M. caesia*, and *M. laurina* had the ability to act as a larvacide and *M. odorata*, *M. laurina*, and *M. indica* inhibited the hatching of *A. aegypti* eggs. Various compounds are responsible for the biological activity of this essential oil. These compounds can jointly or individually contribute to producing larvicidal activity and inhibiting egg hatching [31]. One plant species may contain substances that have different activities; for example, *Cinnamomum impressicostatum* exhibits antibacterial [32], antifungal [33], insecticidal [34], and larvicidal [35] activity. The limitation of this research is that it is only at the preliminary testing stage in the laboratory and requires further testing in the field for community applications.

## Conclusion

The activity of extracts of *M. casturi*, *M. caesia*, *M. laurina*, *M. odorata*, and *M. laurina* was shown to be capable of controlling dengue vectors, especially at the egg and pre-adult stages. It should be noted that the availability of plants that are easy to find and the availability of leaves every year add value to this plant extract. Therefore, this plant extract can be applied for integrated vector control. Further studies on side effects and the effects of the plant on non-target organisms, alongside field evaluations, should be performed.

## Authors’ Contributions

NM: Conception of the study, extraction of simplicia, analyzed the data, and drafted the manuscript. MRR: Conception of the study, analyzed the data, and drafted the manuscript. DS: Extraction of simplicia, analyzed the data, and drafted the manuscript. MRSP, NR, and BPA: Drafted and revised the manuscript. All authors have read and approved the final manuscript.
